# Effects of 3D-Printed Polycaprolactone/β-Tricalcium Phosphate Membranes on Guided Bone Regeneration

**DOI:** 10.3390/ijms18050899

**Published:** 2017-04-25

**Authors:** Jin-Hyung Shim, Joo-Yun Won, Jung-Hyung Park, Ji-Hyeon Bae, Geunseon Ahn, Chang-Hwan Kim, Dong-Hyuk Lim, Dong-Woo Cho, Won-Soo Yun, Eun-Bin Bae, Chang-Mo Jeong, Jung-Bo Huh

**Affiliations:** 1 Department of Mechanical Engineering, Korea Polytechnic University, 237 Sangidaehak-Ro, Siheung-Si, Gyeonggi-Do 15073, Korea; happyshim@kpu.ac.kr (J.-H.S.); thmim123@naver.com (D.-H.L.); wsyun@kpu.ac.kr (W.-S.Y.); 2 Research Institute, T&R Biofab Co., Ltd., 237 Sangidaehak-Ro, Siheung-Si, Gyeonggi-Do 15073, Korea; jywon@tnrbiofab.com (J.-Y.W.); gsahn@tnrbiofab.com (G.A.); chkim@tnrbiofab.com (C.-H.K.); 3 Department of Prosthodontics, Dental Research Institute, Institute of Translational Dental Sciences, BK21 PLUS Project, School of Dentistry, Pusan National University, 49 Pusan University-Ro, Yangsan-Si, Gyeongsangnam-Do 50612, Korea; dentizen1201@nate.com (J.-H.P.); say0739@daum.net (J.-H.B.); 0228dmqls@hanmail.net (E.-B.B.); cmjeong@pusan.ac.kr (C.-M.J.); 4 Department of Mechanical Engineering, Pohang University of Science and Technology (POSTECH), 77 Cheong-Am-Ro, Nam-Gu, Pohang-Si, Gyeongsangbuk-Do 37673, Korea; dwcho@postech.ac.kr

**Keywords:** 3D printing, animal study, collagen membrane, guided bone regeneration (GBR), membrane, polycaprolactone (PCL), β-tricalcium phosphate (β-TCP)

## Abstract

This study was conducted to compare 3D-printed polycaprolactone (PCL) and polycaprolactone/β-tricalcium phosphate (PCL/β-TCP) membranes with a conventional commercial collagen membrane in terms of their abilities to facilitate guided bone regeneration (GBR). Fabricated membranes were tested for dry and wet mechanical properties. Fibroblasts and preosteoblasts were seeded into the membranes and rates and patterns of proliferation were analyzed using a kit-8 assay and by scanning electron microscopy. Osteogenic differentiation was verified by alizarin red S and alkaline phosphatase (ALP) staining. An in vivo experiment was performed using an alveolar bone defect beagle model, in which defects in three dogs were covered with different membranes. CT and histological analyses at eight weeks after surgery revealed that 3D-printed PCL/β-TCP membranes were more effective than 3D-printed PCL, and substantially better than conventional collagen membranes in terms of biocompatibility and bone regeneration and, thus, at facilitating GBR.

## 1. Introduction

Guided bone regeneration (GBR) is the most common method used for treating bone defects in implant dentistry, and many studies have reported it produces satisfactory results [1,2,3,4]. GBR provides a means of inducing new bone formation without perturbing soft tissues. This technique requires that a membrane be placed above a bone defect site to inhibit fibroblast influx from adjacent epithelium and connective tissue, and to maintain a space for bone regeneration, which involves the infiltration of blood vessels from adjacent old bone and the differentiation and proliferation of osteoblasts [5,6,7]. Membranes play an important role in GBR and, for this reason, much effort has been spent on developing better GBR membranes. The requirements of an ideal GBR membrane include biocompatibility, cell occlusion, tissue integration, space-maintaining ability, and clinical manageability [8].

Various materials have been used to fabricate GBR membranes [9], which are generally classified as non-resorbable or resorbable. Non-resorbable membranes have the advantage of excellent space-maintaining ability, but are used only for specific indications due to the need for a second surgery to remove them and a risk of infection caused by a high rate of membrane exposure [9,10]. Resorbable membranes are being increasingly used in clinical practice as their limitations have been largely addressed. These include low space-maintaining ability due to weak mechanical properties, and rapid degradation and absorption [11,12]. Recent, studies have focused on improving the space-maintaining abilities and mechanical properties of resorbable membranes using different fabrication methods and synthetic biodegradable materials [13,14,15]. Of the various fabrication methods used, in combination with synthetic biodegradable materials, three-dimensional (3D) printing enables resorbable membranes to be made without toxic solvents and allows membrane thicknesses, pore sizes, and shapes to be easily adjusted to create favorable environments for cells. 

In a previous study, a PCL/PLGA/β-TCP membrane was fabricated from an intimate mix of polycaprolactone (PCL), poly lactic-co-glycolic acid (PLGA), and beta-tricalcium phosphate (β-TCP) using a multi-head deposition system (an extrusion-based 3D-printing technology) [16,17,18]. An evaluation of the membranes produced in a rabbit calvarial defect model confirmed their superior abilities to promote bone regeneration [16]. Furthermore, when PCL/PLGA/β-TCP and titanium mesh membranes (a typical non-resorbable membrane) were applied to peri-implant defects in a beagle mandible, the PCL/PLGA/β-TCP membrane was found to be non-inferior in terms of new bone formation and osteointegration [17], and the same was found when PCL/PLGA/β-TCP and collagen membranes (a typical resorbable membrane) were compared [18]. These results confirmed the potential of PCL/PLGA/β-TCP membranes for GBR, but PLGA has also been reported to induce an inflammatory response because of the acidic byproduct and toxins produced during its degradation process [19,20,21].

PLGA is a copolymer of polylactic acid (PLA) and polyglycolic acid (PGA) and has been approved by the U.S. Food and Drug Administration (FDA). PLGA is a widely-used biomaterial and is used to produce biodegradable implants and drug carriers due to its excellent biocompatibility, biodegradability, and process ability [22,23]. In previous studies, PLGA, which has a high elastic modulus, was mixed with PCL and β-TCP to enhance membrane strength [19,20,21]. Therefore, this study was undertaken to evaluate the efficacy of PCL/β-TCP (4:1 ratio by weight) membranes fabricated by 3D printing and to compare them with PCL and commercially-available collagen membranes.

## 2. Results

### 2.1. Mechanical Properties of PCL and PCL/β-TCP Membranes 

**Figure 1:**
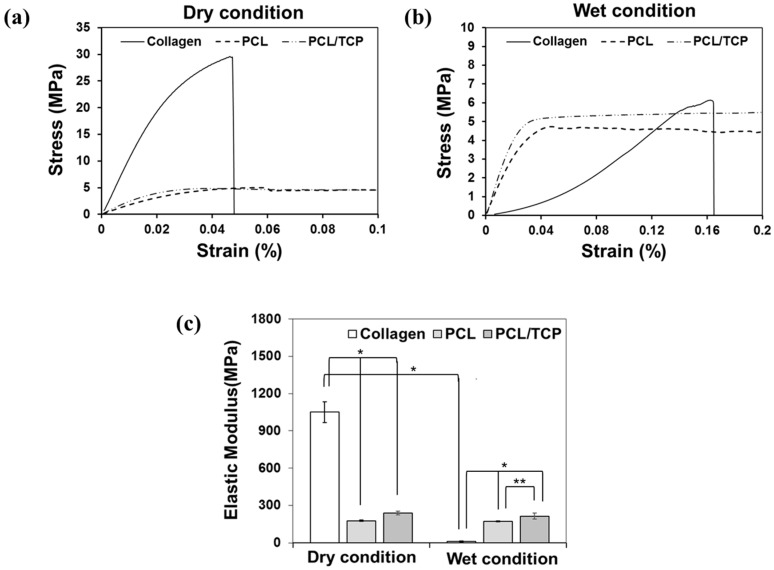
(**a**,**b**) Stress-strain curves of collagen, polycaprolactone (PCL), and polycaprolactone/β-tricalcium phosphate (PCL/β-TCP) membranes under dry and wet conditions; (**c**) Elastic moduli of collagen, PCL, and PCL/β-TCP membranes under dry and wet conditions. (** *p* < 0.01, * *p* < 0.05).

Membrane tensile testing was performed under dry and wet conditions. The results of the stress-strain curves obtained are shown in Figure 1a,b. The tensile stress of dry collagen membranes was significantly higher than that of PCL or PCL/β-TCP membranes. However, when wet, the tensile stresses of all membranes were similar. Membrane elastic moduli were calculated from stress-strain curves (Figure 1c). When dry, the elastic modulus of collagen membranes (1050 MPa) were significantly higher than those of PCL (175 MPa) and PCL/β-TCP (239 MPa) membranes (*p* < 0.001) but, when wet, the elastic moduli of PCL (171 MPa) and PCL/β-TCP (213 MPa) membranes were significantly higher than that of collagen membranes (12 MPa) (*p* < 0.001). Furthermore, PCL/β-TCP membranes had a slightly higher elastic modulus than PCL membranes (*p* < 0.05). These results show that the mechanical strength of collagen is significantly reduced under wet conditions, whereas PCL and PCL/β-TCP membranes were relatively unaffected (Table 1).

### 2.2. In Vitro Results

**Figure 2:**
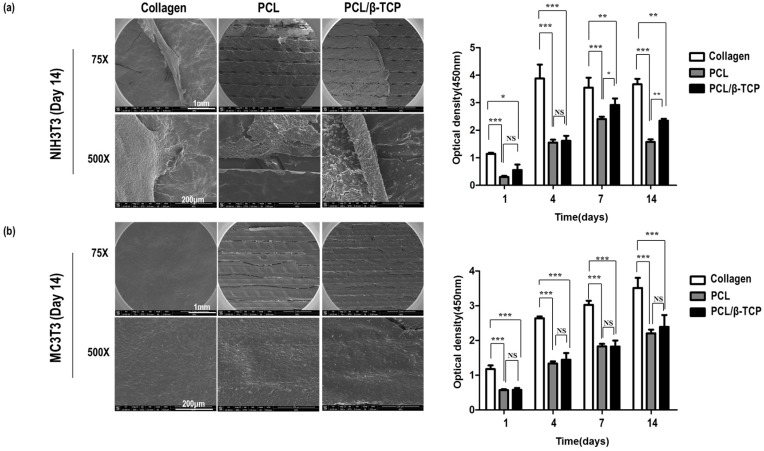
Scanning electron microscopy (SEM) and cell counting kit-8 (CCK-8) assays of NIH3T3 (**a**) and MC3T3 (**b**) cells on collagen, PCL, and PCL/β-TCP membranes showing that attached cells grew well. (*** *p* < 0.001, ** *p* < 0.01, * *p* < 0.05, NS = No significant difference).

A CCK-8 assay and SEM imaging were used to investigate the proliferations and morphologies of NIH3T3 (a mouse fibroblast cell line) and MC3T3-E1 (a mouse pre-osteoblast cell line) cells on collagen, PCL, and PCL/β-TCP membranes (Figure 2). CCK-8 assay results showed that initial adhesion was approximately twice as high on collagen as on PCL or PCL/β-TCP, though the optical density of cells on PCL and PCL/β-TCP membranes was increased with time. However, the optical density of NIH3T3 cells on PCL and PCL/β-TCP decreased after day 7, whereas MC3T3-E1 cells continued to grow on the three different membranes up to day 14. SEM imaging showed this reduction in NIH3T3 cells was due to cell aggregation on the membranes and detachment on day 14. This detachment of NIH3T3 cells was also partially observed on collagen, though much less so than that observed on PCL or PCL/β-TCP. These results suggest that the PCL/β-TCP membrane might be useful for GBR, as it promoted the proliferation of pre-osteoblasts and inhibited fibroblast ingrowth.

**Figure 3:**
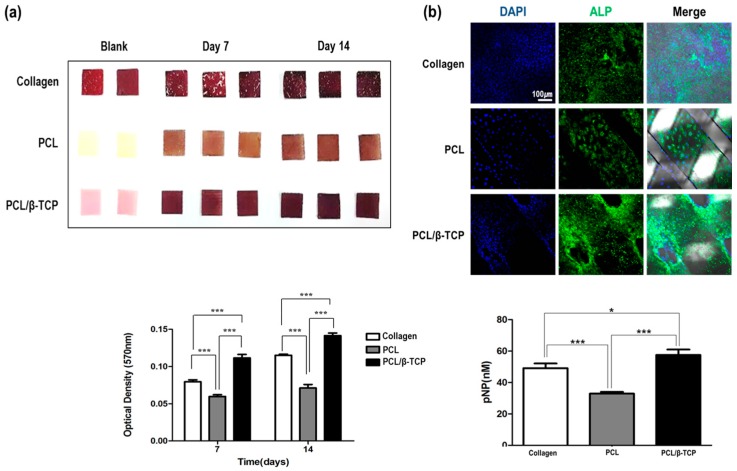
Alizarin red S (**a**) and alkaline phosphatase (ALP) (**b**) staining showed osteogenic differentiation on PCL/β-TCP membranes was greater than on collagen membranes. (*** *p* < 0.001, * *p* < 0.05).

Osteogenic differentiation was examined using MC3T3-E1cells, to check the extent of osteogenesis on the three membrane types (Figure 3). Extents of differentiation were analyzed by alizarin red S staining and quantitated using 10% cetylpyridinium chloride on days 7 and 14. On day 7, the extent of osteogenic differentiation on PCL/β-TCP was 1.5 times higher than that observed on collagen and this difference was maintained until day 14. Regarding early markers of osteogenesis, on day 7, the expression of alkaline phosphatase (ALP) on PCL/β-TCP was higher than on collagen or PCL. Quantitation using p-nitro phenyl phosphate (pNPP) confirmed the higher expression of ALP on PCL/β-TCP.

These in vitro results demonstrate the potential usefulness of the PCL/β-TCP membrane as a GBR membrane, which exhibited cell compatibility, inhibition of external tissue ingrowth, and the promotion of osteogenic differentiation.

### 2.3. In Vivo Results

#### 2.3.1. Clinical Findings

All animals survived and the 18 defects healed without any problems. Membrane exposure and separation were not observed at the end of the study. No complication was encountered in any of the three groups at the end of the study, and clinical difference was not observed.

#### 2.3.2. Volumetric Analysis by Micro-Computed Tomography

**Figure 4:**
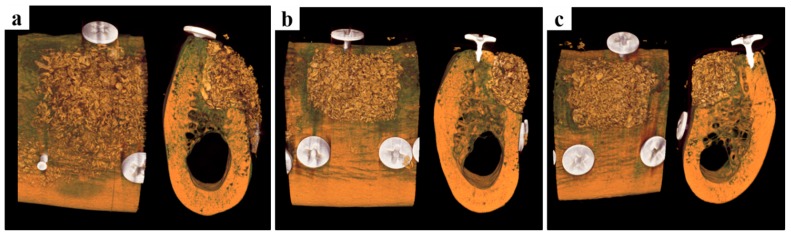
Micro-computed tomography images. (**a**) The collagen group, (**b**) the PCL group, and (**c**) the PCL/β-TCP group. The collagen group exhibited the common alveolar ridge shape, but the PCL and PCL/β-TCP groups maintained a significantly augmented alveolar ridge shape to the lateral side.

3D micro-CT images are presented in Figure 4. In the collagen group, bone graft materials maintained the common alveolar ridge shape. On the other hand, in the PCL and PCL/β-TCP groups maintained an augmented alveolar ridge shape to the lateral side of the alveolar ridge shape. Space maintenance by barrier materials plays a key role in bone augmentation and, thus, these results suggest bone regeneration would be greater in the PCL and PCL/β-TCP groups than in the collagen group. Volumetric measurements are summarized in Table 2. The PCL/β-TCP group showed non-significantly more new bone formation than the other two groups (*p* = 0.350). All three groups had similar remaining bone substitute volumes (mm^3^, *p* = 0.923). In terms of non-mineralized tissue volumes (mm^3^) the three groups were similar (*p* = 0.930).

#### 2.3.3. Histologic Findings

**Figure 5:**
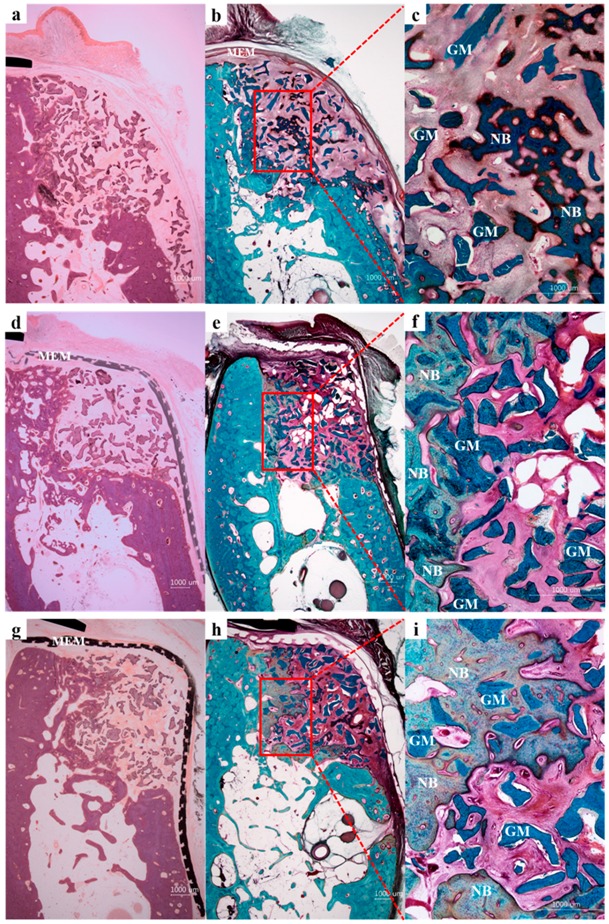
Histological sections of the collagen (**a**–**c**), PCL (**d**–**f**), and PCL/β-TCP (**g**–**i**) groups. NB, new bone; GM, graft material; MEM, membrane. Hematoxylin and eosin (**a**,**d**,**g**) stain and Goldner Trichrome stain (**b**,**c**,**e**,**f**,**h**,**i**); original magnifications 12.5× for (**a**,**b**,**d**,**e**,**g**,**h**) and 50× for (**c**,**f**,**i**)).

At eight weeks after implantation, no membrane exposure or loss was observed in the PCL or PCL/β-TCP groups and, presumably due to the presence of membranes, bone graft materials were well-preserved in defects. However, in the collagen group, the membrane was not in close contact with the lower part of the buccal bone, and floated slightly, indicating a space was present, and as might be expected, some bone graft loss was observed. On the other hand, in the PCL and PCL/β-TCP groups, membranes were closely attached to the buccal bone. In most specimens, new bone was observed around bone graft materials. In the collagen group (Figure 5a–c), small amounts of new bone were observed in defects. On the other hand, in the PCL (Figure 5d–f) and PCL/β-TCP groups (Figure 5g–i), large amounts of new bone were observed. Furthermore, bone graft materials were augmented to the buccal lateral side of the alveolar ridge, that is, they retained the shape of the membranes as formed by the surgeon.

#### 2.3.4. Histometric Analysis

Histometric measurements are summarized in Table 3 and Table 4. Areas of tissue compartments within membrane-protected bone defects were analyzed for three membrane systems using new bone plus bone substitute percentages (NIB, %) and soft tissue plus bone substitute percentages (SIB, %) in membrane-enclosed defect areas in vertical sections. The PCL/β-TCP group had significantly higher NIB% values than the other two groups (*p* < 0.001) but significantly lower SIB% levels (*p* < 0.05). In terms of soft tissue plus bone substitute percentages (SEB, %), the collagen group showed significantly higher levels than the other groups (*p* < 0.001). With regard to horizontal width measurements of ridge augmentation, bone gain at the 75%, 50%, and 25% defect height levels were significantly different in the three groups, and the PCL/β-TCP group had significantly higher values than the collagen or PCL groups (*p* < 0.01).

## 3. Discussion

In the present study, GBR membranes were fabricated using an extrusion-based 3D printer, which has been the subject of several studies. [17,18,24,25] A strong merit of extrusion-based 3D printing systems is that they enable composited biomaterials, such as, PCL, PLGA, PLA, PGA, and their blends to be used. In addition, powder materials, particularly those with thermal resistance, can be mixed with these thermoplastic polymers. Indeed, blends of PCL/PLGA [24], PCL/PLGA/powdered TCP [26] and PCL/PLGA/powdered antibiotics [18] have been shown to be potential candidates for engineering tissue scaffolds by 3D printing. 

The membranes used for GBR can be classified as barrier or support membranes. The barrier membranes typically used in clinical practice are collagen membranes, and the support membranes are typically made of titanium. Although titanium membranes have excellent space-maintaining abilities, they are non-absorbent and their porous structures cannot be easily controlled. For this reason, we developed a PCL/PLGA/TCP membrane with the aim of replacing the titanium support membrane. Considering the strength of titanium, we used a PCL/PLGA/TCP blend, in which the role of the PLGA was to enhance membrane stiffness [17,18]. However, the acidic products generated during the degradation of PLGA have been reported to cause rapid swelling and inflammatory response in vivo, and PLGA, including membranes, have been reported to fail structurally because of their rapid degradation rates and inflammatory response [27,28,29,30,31]. Therefore, we decided to exclude PLGA, and focused on PCL/β-TCP membranes for GBR. 

PCL is known as an effective biodegradable synthetic polymer in the tissue engineering field, and has sufficient elasticity to prevent early membrane fracture [32]. However, its cell affinity is lower than those of other polymers because of its hydrophobicity [27,33], and its degradation rate is much slower than that of bone reconstruction [34]. The scaffold degradation rate is an important consideration, as it is closely related to cell vitality and growth [35]. Scaffolds should degrade slowly enough to maintain space during initial new bone growth, but rapidly enough to provide space for new bone formation. Accordingly, many attempts have been made to increase the degradation rate of PCL. In the present study, β-TCP was used to increase the degradation rate of PCL, as it has been shown β-TCP is biodegraded in vivo in 3–6 months, whereas PCL biodegradation takes more than 12 months. 

Furthermore, the PCL/β-TCP blend was shown to have better physical, biological, and mechanical properties than PCL alone in this study. Ceramic β-TCP has excellent osteoconductivity and biocompatibility and, thus, is widely used in orthopedics and dentistry [36], and the incorporation of TCP into PCL scaffolds [26,37] and membranes [16] can increase compressive strength and surface roughness and, thus, enhance cell and tissue integration. 

The role of the membrane is to prevent fibroblast invasion and aid bone formation. In this study, the PCL/β-TCP membrane acted as a barrier and, simultaneously, β-TCP releases Ca^2+^ to aid bone formation. As a role to enhance bone regeneration, preosteoblast cell adhesion and differentiation were evaluated. If the surrounding tissues are not adhered to the membrane at the time of insertion, the body can represent a foreign body reaction. Therefore, it is important to determine the relationship with the surrounding tissue. This in vitro testing using NIH3T3 cells (mouse fibroblasts) and MC3T3-E1 cells (mouse preosteoblasts) showed that PCL and PCL/β-TCP membranes were comparable to collagen membranes in terms of cell proliferation and osteogenic differentiation (Figure 2 and Figure 3). PCL/β-TCP membranes had excellent cell affinity and mechanical properties and were flexibility enough to manage irregular bone defects. 

In the dental field, collagen membranes are regarded as the gold standard for GBR, due to their excellent biocompatibility, low immunogenicity, and optimal biodegradability. In the present study, collagen membranes demonstrated excellent in vitro and in vivo biocompatibility, but they also exhibited rapid loss of space-making ability under wet conditions. In fact, the fiber-like state of dry collagen membranes rapidly became gel-like after wetting. The elastic modulus of dry collagen membranes (1050.2 ± 84.10 MPa) was superior to those of PCL (175.5 ± 5.40 MPa) and PCL/TCP (238.9 ± 15.70 MPa) membranes (Figure 1). However, the elastic modulus of collagen membranes rapidly decreased from 1050.2 ± 84.10 MPa to 12.0 ± 3.90 MPa when dry membranes were exposed to a wet environment. Furthermore, this in vivo experiment showed that after collagen membranes had absorbed water, they were weakened to the extent that they were no longer able to maintain their space-making ability. On the other hand, the elastic moduli of PCL and PCL/β-TCP membranes were similar under dry and wet conditions. The hydrophobic nature of PCL means that its uptake of water occurs slowly [38] and, thus, the mechanical properties of PCL and PCL/β-TCP membranes do not change appreciably after several days of exposure to water, which indicates PCL and PCL/β-TCP membranes are likely to retain their space-making abilities in the presence of saliva or blood.

In the clinical situation, the GBR procedure is mainly performed to augment bone growth at defect sites. In the present study, box-type defects of 7 mm × 5 mm × 5 mm were prepared in mandibles to mimic large bone defects, and then treated by GBR [16]. The three dogs were sacrificed at eight weeks after surgery, which was determined based on considerations of bone turnover and the bone regeneration rate, for volumetric and histomorphometric analysis. Micro-CT analysis showed volumetric parameters were not significantly better in the PCL/β-TCP group, and that this group maintained an augmented alveolar ridge shape to the lateral side. Space maintenance by bone graft materials plays an important role in bone augmentation. Jiang et al. [39] reported facial pressure affected new bone formation in defect areas and that space-maintaining ability was critical for new bone formation. 

Histologic findings showed PCL and PCL/β-TCP membranes were attached to the buccal bone and that bone graft materials were augmented to the buccal lateral side in accord with the membrane shape determined by the surgeon, whereas in the collagen group, membranes floated away from the buccal bone and, thus, graft materials were scattered. In clinical practice, GBR membranes are left in place for up to six months, which is the optimal time for bone regeneration [40]. However, in our histologic study, substantially intact PCL and PCL/β-TCP membranes were observed eight weeks after implantation. Accordingly, further study is required to determine the degradation rate of PCL/β-TCP membranes.

In the histometric analysis, we analyzed areas of tissue compartments within membrane-protected bone defects and horizontal bone gains at different defect levels. In terms of NIB% and SEB% values, the PCL/β-TCP group showed significantly better results than the other two groups (*p* < 0.001). Furthermore, horizontal measurements of ridge augmentation showed bone gains at the 75%, 50%, and 25% level results were significantly better in the PCL/β-TCP group (*p* < 0.01). These results show β-TCP incorporation aided bone regeneration, presumably because of its osteoconductivity. In addition, the use of a PLGA and ß-TCP blend was reported to promote bone regeneration to an extent like that of β-TCP [41].

3D printing technology enables PCL/β-TCP membranes to be prepared quickly and economically in diverse shapes, thicknesses, pore sizes, pore geometries, and porosities. In addition, its mechanical properties and degradation rates can be modified by changing the PCL: β-TCP ratio [17,32]. In addition, they can be tailor-made for patients using CT scan data, and drugs or growth factors can be incorporated [42,43]. In this study, 3D-printed resorbable PCL/β-TCP membranes were found to provide high-stability GBR membranes with excellent new bone formation abilities, and to offer a feasible alternative to collagen membranes. Further studies are required to investigate different uses and optimize PCL: β-TCP ratios.

## 4. Materials and Methods 

### 4.1. Fabrication of PCL and PCL/β-TCP Membranes Using 3D Printing Technology 

#### 4.1.1. Polymers and Polymer Blending 

PCL (19561-500G, Polysciences Inc., Warrington, PA, USA) and β-TCP (average diameter: 100 nm, Berkeley Advanced Biomaterials Inc., Berkeley, CA, USA) were used to fabricate PCL and PCL/β-TCP membranes. PCL (0.4 g) granules were melted in a glass container for 10 min at 110 °C, and powdered β-TCP (0.1 g) was added to the PCL melt.

#### 4.1.2. Fabrication of PCL and PCL/β-TCP Membranes

Membranes were prepared from PCL and PCL/β-TCP melts, which were placed in the steel syringe of the multi-head deposition system (MHDS) and maintained at a temperature of 110 °C for dispensing. The MHDS 3D printing system had four extrusion heads, which were controlled with respect to temperature, pneumatic pressure, and motion [16]. Melts were dispensed from the nozzle at 110 °C and 500 kPa. The PCL and PCL/β-TCP membranes were composed of four layers and were fabricated layer-by-layer to produce 3D-multilayer mesh-type structures similar to that of collagen membranes. The 3D-printed membranes had a triangular pore structure. The membranes produced were 10 mm × 10 mm × 0.32 mm, and the mean line widths and pore sizes of the struts were 500 μm and 250 μm, respectively. 

#### 4.1.3. Mechanical Testing of Collagen, PCL, and PCL/β-TCP Membranes

Mechanical tensile tests were conducted using a single-column tensile tester (Instron Co., Norwood, MA, USA). The test conditions were as described in ISO527 (tensile test on plastics), and specimen dimensions were based on Specimen Type 4 of ISO527-3. In detail, a standardized tensile specimen having two shoulders and a gauge in between was fabricated in which the overall length, gauge length, width of the shoulder, and width of the gauge were 152, 50, 38, and 25 mm, respectively. Under dry and wet conditions (after soaking in a medium for 18 h), loads and displacements were measured at a constant cross-head speed of 5 mm/min.

### 4.2. In Vitro Analysis

#### 4.2.1. Cell Culture and Proliferation Assay

NIH3T3 (mouse fibroblasts, ATCC #CRL-1658) and MC3T3-E1 (mouse preosteoblasts, ATCC #CRL-2593) were cultured in Dulbecco modified Eagle’s medium (DMEM) and α-MEM containing 10% fetal bovine serum (FBS) and 1% penicillin and streptomycin (all from Invitrogen, Carlsbad, CA, USA). To aid cell seeding, membranes (all 10 mm × 10 mm × 0.32 mm) were pre-wetted in culture medium for 2 h and then exposed to UV for 30 min. Cells were seeded at a density of 3 × 10^5^ cells per membrane and incubated in a humidified 5% CO_2_ atmosphere at 37 °C. Rates of proliferation on the membranes were quantified using a Cell Counting Kit-8 (CCK-8, Dojindo, Rockville, MD, USA) on days 1, 4, 7, and 14 after seeding.

#### 4.2.2. Osteogenic Differentiation and Alizarin Red Stain Staining

MC3T3-E1-seeded membranes were cultured in osteogenic medium (α-MEM containing 20% FBS, 10–8 M dexamethasone, 0.2 mM ascorbic acid, 10 mM β-glycerol phosphate (Sigma Aldrich, Saint Louis, MO, USA) and 1% penicillin/streptomycin. Samples were fixed with 4% paraformaldehyde on days 7 or 14 and stained with 2% alizarin red (pH 4.2) for 5 min at room temperature. To estimate the amounts of calcium deposition, membranes were soaked in 10% cetylpyridinium chloride for 10 min at room temperature and optical densities was measured at 570 nm using a microplate reader (Epoch, BioTek, Winooski, VT, USA).

#### 4.2.3. Scanning Electron Microscopy (SEM)

Cell morphologies on membranes were determined by high-resolution SEM at an acceleration voltage of 10 kV (Nova NanoSEM 450, FEI, Hillsboro, OR, USA).

### 4.3. In Vivo Analyses

#### 4.3.1. Experimental Animals

Three healthy laboratory bred beagles were used in the study. These animals were approximately three years old and weighed 13–15 kg. All experiments were conducted according to the guidelines issued by Chonnam National University Animal Hospital, and animal selection, management, and surgical procedures were approved by the Ethics Committee on Animal Experimentation of Chonnam National University (CNU IACUC-TB-2013-10 (11 May 2013)).

#### 4.3.2. Experiment Design and Time Schedule

Three box-type bone defects were made on each side of the dog mandibles for ridge augmentation. A defect measuring tool (length: 7 mm, height: 5 mm. and depth: 5 mm) was used to standardize defect dimensions. A total of 18 bone defects were created in the three dogs (six/dog) and the following treatments were randomly applied:

- Collagen group: Collagen membrane (GCM2030, GENOSS, Suwon, Korea) with deproteinized bovine bone grafting material (Bio-Oss, Geistlich Biomaterials, Wolhusen, Switzerland).
- PCL group: PCL membrane with the same deproteinized bovine bone grafting material.
- PCL/β-TCP group: PCL/β-TCP membrane with the same deproteinized bovine bone grafting material.

#### 4.3.3. Surgical Procedures

**Figure 6:**
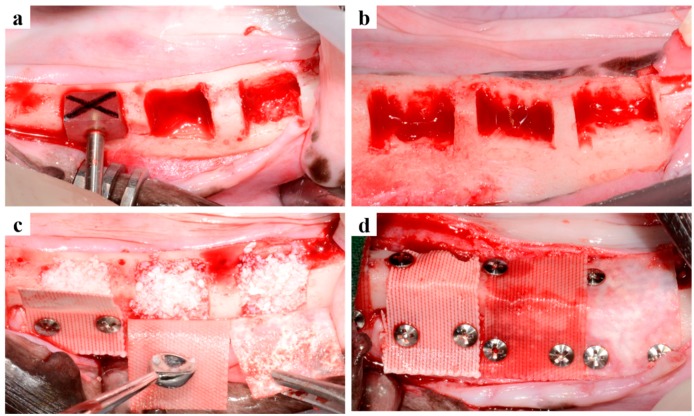
Surgical procedures. (**a**) The alveolar ridge crest was flattened and a defect measuring tool was used to produce same sized defects; (**b**) Six box-type defects (length: 7 mm, height: 5 mm, depth: 5 mm) were prepared per dog; three on right and left sides; (**c**) Defects were filled with particle-type graft material, and membranes were placed randomly on defects; (**d**) Titanium pins were used for membrane fixation.

Surgery was performed in two stages (Figure 6). Initially, surgery was performed to create edentulous ridges. General anesthesia was performed with atropine (Dai Han Pharm Co., Seoul, Korea) and an intramuscular injection of xylazine (Rompun, Bayer Korea Co., Seoul, Korea), and maintained by isoflurane inhalation (Choongwae Co., Seoul, Korea). In addition, local infiltration anesthesia at surgical sites was performed with 1 mL of 2% lidocaine HCL and 1: 100,000 epinephrine (Yu-Han Co., Gunpo, Korea). All premolars and first molars were bilaterally extracted, and after extraction, surgical sites were sutured with 4-0 Vicryl^®^ (Johnson and Johnson, New Brunswick, NJ, USA).

The second surgery for GBR was performed eight weeks after initial surgery. Anesthesia was performed in the same manner as that described above for initial surgery. A mid-crestal incision was made at surgical sites (first premolars to first molars), and vertical incisions were placed. Mucoperiosteal flaps were elevated and alveolar ridge crests were flattened. Box-type bone defects (length: 7 mm, height: 5 mm, depth: 5 mm) were created using the defect measuring tool (Figure 6a,b) while saline solution was being supplied. The particle type graft material (Bio-oss, Geisthlich Biomaterials, Wolhusen, Switzerland) was dispensed (~0.1 g) using a micro spoon (Karl Hammacher GmbH Co., Cologne, Germany) and dampened with normal saline solution for 5 min (Figure 6c). The box defects were then filled with the particle-type graft material, and the collagen (GCM2030, GENOSS, Suwon, Korea), PCL, or PCL/β-TCP membranes were randomly positioned on the defects. To enhance stability titanium pins (Dentium Co., Seoul, Korea) were used for membrane fixation (Figure 6d). The flaps were then carefully released and sutured with 4-0 Vicryl^®^ (Johnson and Johnson, New Brunswick, NJ, USA).

#### 4.3.4. Post-Operative Care and Sacrifice

Antibiotics were injected immediately after the second surgery and 48 h later. Plaque was controlled by daily flushing of the oral cavity until sacrifice. The animals were kept on a soft food diet for the entire study period. Eight weeks after the second surgery, animals were sacrificed by injecting concentrated sodium pentobarbital (Euthasol, Delmarva Laboratories Inc., Midlothian, VA, USA). Mandibles were harvested and immediately immersed in neutral-buffered formalin (Sigma Aldrich, St. Louis, MO, USA).

#### 4.3.5. Micro-Computed Tomography 

**Figure 7:**
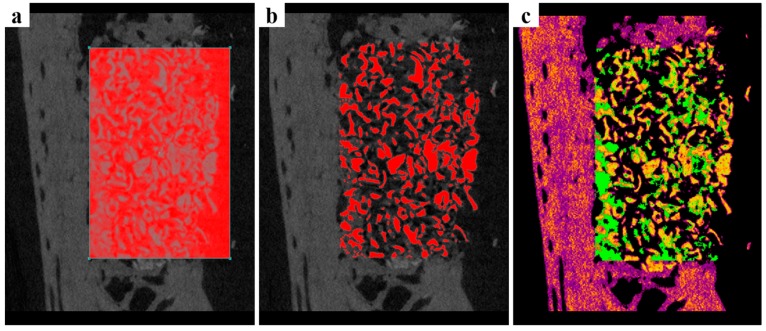
Micro-CT images. (**a**) The region of interest; length: 7 mm, height: 5 mm, depth: 5 mm; (**b**) Bone graft materials shown in red; (**c**) New bone shown in green with bone graft materials in yellow.

Micro-CT was used to evaluate the new bone formation in the defects. Harvested mandibles were wrapped with Parafilm M^®^ (Bemis Company, Inc., Neenah, WI, USA) to prevent drying during scans, which were performed using the following conditions: 130 kV energy, 60 μA intensity, and a 7.10 μm-pixel resolution; on a Bruker-micro CT using SkyScan-1173 version 1.6 (Bruker; Kontich, Belgium). Reconstruction was performed using Nrecon version 1.6.10.4 (Bruker). ROIs were placed on box defects (length: 7 mm, height: 5 mm, depth: 5mm; Figure 7), and the following parameters were calculated:

- New bone volume (NBV; mm^3^): Volume occupied by new bone.
- Remaining bone substitute volume (RBV; mm^3^): Volume occupied by remaining bone substitute.
- Non-mineralized tissue volume (NMV; mm^3^): Volume occupied by non-mineralized tissue.

#### 4.3.6. Histomorphometric Analysis

**Figure 8:**
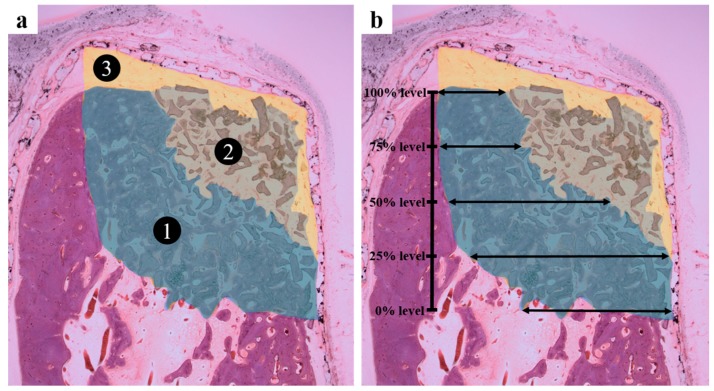
Histometric analysis. (**a**) Tissue compartment areas within bone defect were assessed; (**b**) Horizontal bone growths at different defect heights depths were measured.

After micro-CT scans, harvested mandibles were washed and dehydrated using ethanol, and embedded in Technovit 7200 resin (Heraeus Kulzer, Germany), which was polymerized using a UV embedding system (Kulzer Exakt 520, Exakt, Norderstedt, Germany). Samples were then sectioned (~40 μm) at defect centers using the Exakt diamond cutting system (Exakt 300 CP, Germany). To observe bone regeneration, sections were stained with H and E and Goldner Trichrome, and images were captured under a light microscope (BX51, Olympus, Japan) equipped with a CCD digital camera (Spot Insight 2 MP scientific CCD digital camera system; Diagnostic Instruments, Inc., Ann Arbor, MI, USA). I-Solution ver. 8.1 (IMT I-Solution, Inc., Coquitlam, BC, Canada) was used for the histometric analysis of the captured images. The area of interest (AOI) was defined by the box defect (height: 5 mm, depth: 5 mm; Figure 8). The following parameters were calculated within the AOIs [44]:

- New bone including bone substitute (NIB%; Figure 8a ①): Area occupied by new bone and bone substitute/AOI × 100 (%)
- Soft tissue including bone substitute (SIB%; Figure 8a ②): Area occupied by soft tissue and bone substitute/AOI × 100 (%)
- Soft tissue excluding bone substitute (SEB%; Figure 8a ③): Area occupied by soft tissue/AOI × 100 (%)
- Horizontal bone gain at different defect levels (mm; Figure 8b): Distances were measured from the inside of the residual lingual cortex to the most buccal aspect of new bone formation at height levels of 0%, 25%, 50%, 75%, and 100% of the former bone defect.

#### 4.3.7. Statistical Analysis

Statistical analyses were performed using R software ver 3.1.3. (Lucent technologies, Murray Hill, NY, USA) [45]. The treatment group (collagen, PCL, and PCL/β-TCP groups) was set as the independent factor, and the defect number as the random factor. A non-parametric mixed model with post hoc analysis was used to compare group micro-CT findings and histometric parameters.

## 5. Conclusions

In this study, the 3D-printed PCL/β-TCP membrane demonstrated better bone regeneration performance than the collagen membrane for a GBR procedure performed in buccal defects, which indicated the PCL/β-TCP membrane has potential use as a resorbable GBR membrane for the treatment of alveolar bone defects. Furthermore, the observed greater structural stability of the 3D-printed PCL/β-TCP membrane suggests it offers a feasible alternative to collagen membranes for GBR.

## Figures and Tables

**Table 1:** Maximum tensile stresses and elastic moduli of collagen, PCL, and PCL/β-TCP membranes under dry and wet conditions (means ± SDs; *n* = 5).

Group	Maximum Tensile Stress (MPa)		Elastic Modulus (MPa)	
	Dry	Wet	Dry	Wet
Collagen	26.16 ± 5.10	5.10 ± 1.00	1050.2 ± 84.10	12.0 ± 3.90
PCL	4.90 ± 0.15	4.69 ± 0.17	175.5 ± 5.40	171.5 ± 3.80
PCL/β-TCP	5.07 ± 0.40	4.96 ± 0.37	238.9 ± 15.70	213.1 ± 24.70

**Table 2:** Volumetric analysis within region of interest (means ± SDs; *n* = 6; mm^3^).

Group	NBV (mm^3^)	RBV (mm^3^)	NMV (mm^3^)
**Collagen**			
Mean ± SD	25.92 ± 6.97	24.66 ± 9.20	125.14 ± 15.20
Median	27.06	22.65	126.23
**PCL**			
Mean ± SD	27.29 ± 2.19	24.84 ± 5.30	123.58 ± 5.56
Median	27.64	25.59	123.71
**PCL/β-TCP**			
Mean ± SD	29.22 ± 3.11	24.12 ± 5.48	122.37 ± 7.33
Median	28.48	24.48	124.44
*p*	0.350	0.923	0.930

NBV; new bone volume, RBV; remaining bone substitute volume, NMV; non-mineralized tissue volume. No significant differences were observed between the three groups.

**Table 3:** Areas of tissue compartments within membrane-protected bone defect (means ± SDs; *n* = 6; %).

Group	NIB (%)	SIB (%)	SEB (%)
**Collagen**			
Mean ± SD	24.38 ± 7.80	52.05 ± 6.01	23.57 ± 5.32
Median	23.60	53.98	25.88
**PCL**			
Mean ± SD	28.58 ± 5.52	54.59 ± 7.74	16.83 ± 6.30
Median	26.63	54.55	17.64
**PCL/β-TCP**			
Mean ± SD	45.06 ± 12.26	40.44 ± 12.15	14.50 ± 2.19
Median	45.92	41.97	14.89
*p*	<0.001	<0.05	<0.001

NIB; new bone plus bone substitute, SIB; soft tissue plus bone substitute, SEB; soft tissue plus bone substitute.

**Table 4:** Measurements of horizontal ridge augmentation gain at different defect levels (means ± SDs; *n* = 6; mm).

Groups	Bone Gain at 100%	Bone Gain at 75%	Bone Gain at 50%	Bone Gain at 25%	Bone Gain at 0%
**Collagen**					
Mean ± SD	0.53 ± 0.71	1.17 ± 0.67	1.30 ± 0.44	1.70 ± 0.99	5.38 ± 0.93
Median	0.26	1.03	1.20	1.57	5.46
**PCL**					
Mean ± SD	0.58 ± 0.46	1.70 ± 1.00	1.50 ± 0.68	1.31 ± 0.69	5.28 ± 1.81
Median	0.48	1.48	1.38	0.95	6.00
**PCL/β-TCP**					
Mean ± SD	0.76 ± 0.60	3.16 ± 0.99	2.44 ± 0.87	2.99 ± 1.33	4.78 ± 1.24
Median	0.61	3.40	2.22	2.94	4.99
*p*	0.591	< 0.001	< 0.01	< 0.01	0.538

## Abbreviations

PCL	Polycaprolactone
β-TCP	Beta-tricalcium phosphate
GBR	Guided bone regeneration
3D	Three-dimensional
PLGA	Poly lactic-co-glycolic acid
PLA	Polylactic acid
PGA	Polyglycolic acid
FDA	Food and drug administration
ALP	Alkaline phosphatase
pNPP	*p*-nitrophenyl phosphate
FDM	Fused deposition modeling
NIB	New bone including bone substitute
SIB	Soft tissue including bone substitute
SEB	Soft tissue excluding bone substitute
MHDS	Multi-head deposition system
DMEM	Dulbecco modified Eagle’s medium
FBS	Fetal bovine serum
HR-SEM	High resolution-soft tissue excluding bone substitute
ROI	Region of interest
NBV	New bone volume
RBV	Remaining bone substitute volume
NMV	Non-mineralized tissue volume
AOI	Area of interest
